# Traumatic aortic injury: does the anatomy of the aortic arch influence aortic trauma severity?

**DOI:** 10.1007/s00595-016-1443-0

**Published:** 2016-11-17

**Authors:** Jacek Wojciechowski, Lukasz Znaniecki, Kamil Bury, Kamil Chwojnicki, Jan Rogowski

**Affiliations:** 1 Department of Cardiac and Vascular Surgery, Medical University of Gdansk, ul. Dębinki 7, 80-923 Gdansk, Poland; 2 Department of Neurology, Medical University of Gdansk, Gdansk, Poland

**Keywords:** Aorta, Trauma, Stent-graft, Thoracic trauma, Injury

## Abstract

**Purpose:**

Traumatic aortic injury (TAI) is a rare but life-threatening type of injury. We investigate whether the anatomy of the aortic arch influences the severity of aortic injury.

**Methods:**

This is a retrospective study of twenty-two cases treated with TEVAR for TAI in our department from 2009 to 2014. Aortic injury was assessed in accordance with the recommendations of the Society of Vascular Surgery. We measured the aortic arch angle and the aortic arch index, based on the initial angio-CT scan, in each of the analyzed cases.

**Results:**

The mean aortic arch index and mean aortic arch angle were 6.8 cm and 58.3°, respectively, in the type I injury group; 4.4 cm and 45.9° in the type III group; 3.3 cm and 37° in the type IV group. There were substantial differences in both the aortic arch index and the aortic arch angle of the type III and IV groups. A multivariate analysis confirmed that the aortic arch angle was significantly associated with the occurrence of type III damage (OR 1.5; 95% CI 1.03–2.2).

**Conclusions:**

The severity of TAI is influenced by the sharpness of the aortic arch. There is an inverse relationship between the severity of aortic injury and the aortic arch index.

## Introduction

Damage to the aortic isthmus [traumatic aortic injury (TAI)] by blunt chest trauma is the second leading cause of death in trauma patients [1]. This type of trauma is associated with high mortality in the first hours after the injury. Burkhart et al. found that 37% of deaths due to TAI occurred in the first 4 h of admission, and 6% of the patients died consecutively over the next 4 h [2]. Thus, TAI represents a clinical condition that requires prompt action. Endovascular treatment (TEVAR), which is associated with decreased mortality and morbidity, has become an essential tool in the treatment of TAI [3].

Grave aortic injuries often occur in young people. It has been proven that the anatomy of the aorta changes with age [4–6]. Thus, the question arises: is the severity of the injury the result of the nature of the chest injuries suffered by younger patients, or is it associated with the specific aortic anatomy of this group? This question has not been clarified in the literature.

Addressing this issue has practical implications, as the technical problems that are commonly observed in the endovascular treatment of TAI patients are related to the small size of the aorta, and the sharp aortic arch angle. These anatomical factors may be responsible for poor conformation of a stent-graft, the “bird beak” phenomenon, and may lead to late complications (type Ia endoleak or device migration) [7].

The aim of the present study is to evaluate whether there is a relationship between the anatomy of the aorta and the degree of aortic injury.

## Materials and methods

### Patients

The study was conducted retrospectively by analyzing the results of endovascular treatment in patients with TAI due to blunt chest trauma who were treated in our department between 2009 and June 2014. Twenty-nine patients with aortic lesions were treated using TEVAR. We excluded 7 patients from the group who were treated for false aneurysms of the aortic isthmus in which the injury had occurred 2–20 years earlier. The remaining 22 patients with acute TAI were included in the analysis.

The study population included 17 men (77.3%) and 5 women of 21–66 (38.8 ± 13.54) years of age. Twenty-one of the patients were treated in an emergency setting, and one patient was treated in an elective setting. The severity of injury was evaluated according to the Injury Severity Score (ISS) [8] of each patient.

Each of the patients with suspected blunt chest trauma underwent an ECG-gated CT scan, which was performed in the emergency department of our hospital or in the referring hospital. Seven patients were diagnosed with an aortic injury at the referring hospital. The time from injury to TEVAR was approximately 24 h in 5 cases, 5–6 h in 6 cases; the procedure was performed within 3 h after the injury in the remaining cases. In all of the acute cases, the delay was no more than 2 h after admission to our hospital. In one case, the treatment was carried out after 2 months. The case involved a patient with a type I aortic injury, where the injury met the criteria for a “minimal aortic injury.” After two months, a follow-up CT scan of aorta revealed progression into a pseudoaneurysm. The treatment strategy was, therefore, changed from watchful waiting to TEVAR.

In cases in which a diagnosis of aortic isthmus damage was confirmed, the patient was scheduled for endovascular treatment in either the interventional radiology suite (Siemens Artis Zee, Erlanger, Germany), or the operating room using a C-arm (Siemens Arcadis Avantic, Erlanger, Germany).

### Evaluation of the aortic arch

The measurement of aortic arch was made based on DICOM picture reconstruction using the OsiriX DICOM Viewer software program (version 3.9.4; Pixmeo Sàrl, Bernex, Switzerland). Aortic arch reconstruction in 3D MPR was performed for all of the cases. To allow the estimated parameters to be easily applied in the emergency setting, we decided not to use the centerline model. Reconstructions were used for the evaluation of two parameters: the aortic arch index and the aortic arch angle.

The aortic arch index was obtained by measuring the distance from the outer wall of the ascending aorta to the outer wall of the descending aorta on the lesser curvature in the horizontal plane, at the height of the mid-left bronchus. In cases involving large aortic lesions and aneurysmal dilatation of the descending aorta at the level of measurement, the outer wall of the descending aorta was determined by drawing a line between two unaffected points (above and below the lesion), which were placed on unaffected segments of the aorta.

**Fig. 1:**
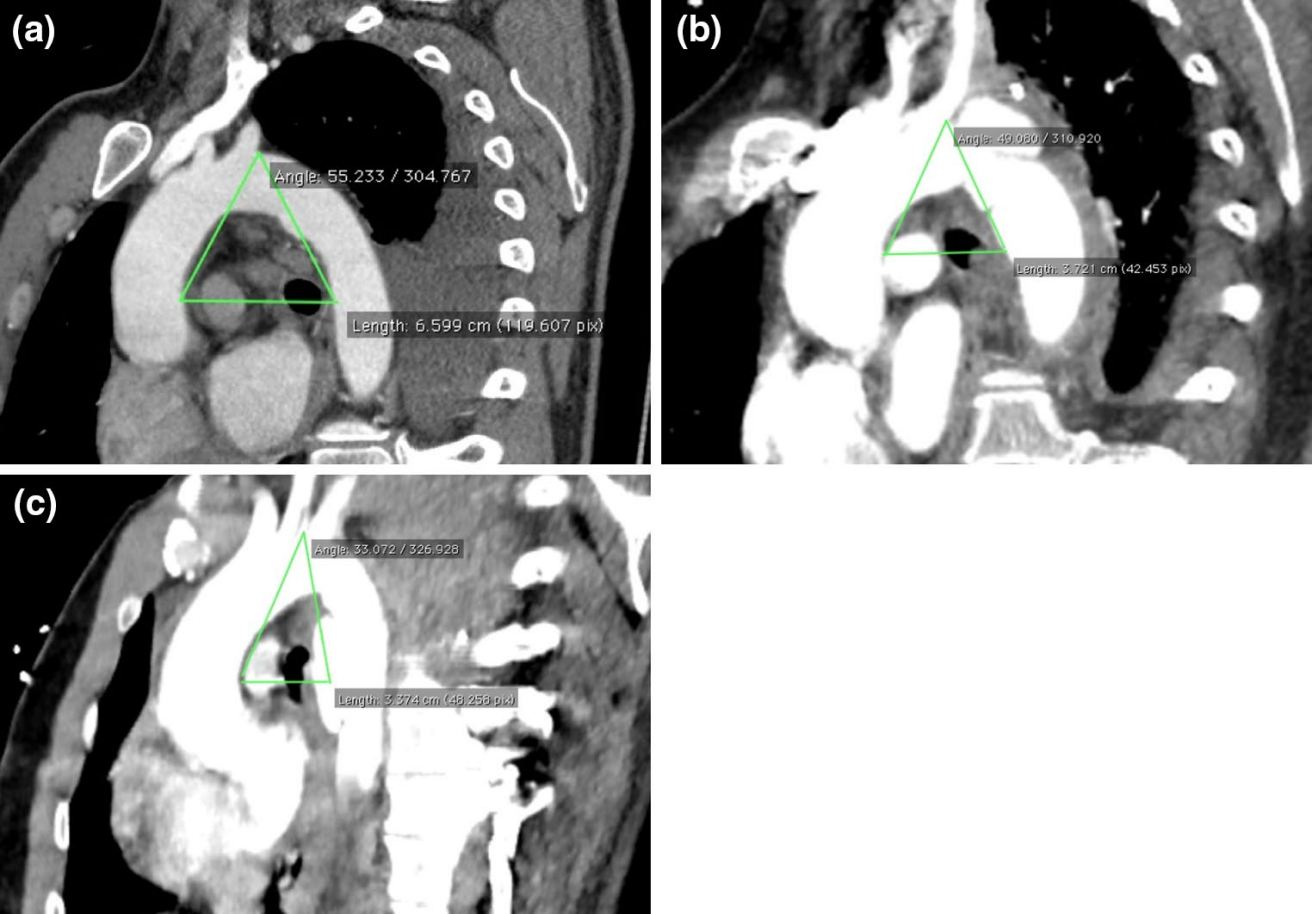
**a** Type I injury. **b** Type III injury. **c** Type IV injury

The aortic arch angle was obtained by measuring the angle created by connecting three points: (1) the outer wall of ascending aorta on the level of mid-left bronchus; (2) the highest point on the outer wall of the aortic arch (greater curvature); (3) the outer wall of the descending aorta at the level of the mid-left bronchus. In cases involving aneurysmal dilatation of descending aorta, the measurement was performed using the above-mentioned method (Fig. 1a–c).

To compare our new parameters to conventional parameters, we also measured the aortic diameter at specified levels. The measurements were performed from centerline models at the levels of the ascending aorta (above the sinus of Valsalva), the aortic arch (before origin of left carotid artery), and the descending aorta (behind the origin of the LSA, at the level of pulmonary artery division and at the level of diaphragm).

The degree of trauma to the aorta was evaluated based on the scale proposed by Azizzadeh et al. in accordance with the SVS recommendations [9, 10].

A Zenith TX2 (Cook Inc., Bloomington, USA) stent-graft was used in 21 (95.4%) cases; in the remaining case (4.6%), an E-vita THORACIC (JOTEC GmBH, Hechingen, Germany) stent-graft was used. The ostium of the LSA was covered in cases in which the seal zone of the healthy aorta was <1 cm.

### Statistical analysis

All calculations were performed using the SAS System software program (v.9.4; SAS Institute, Cary, USA). Continuous variables were expressed as the mean and standard deviation (SD). Categorical variables were expressed as percentages. Fisher’s exact test (categorical values) and the *U* Mann–Whitney test (continuous variables) were used to analyze simple associations. Two-tailed *p* values of <0.05 were considered to indicate statistical significance. A multivariate analysis was performed with a stepwise logistic regression model (odds ratio and 95% confidence limit). The distribution of age, angle and distance were tested with the Shapiro–Wilk and Kolmogorov–Smirnov tests. An ROC curve analysis was performed to evaluate sensitivity and specificity of the aortic arch angle for predicting the occurrence of type III and IV injuries.

## Results

### Clinical outcomes

The early mortality rate was 9.1%. In one case, death was related to abdominal complications after the injury; the direct cause of death was MOF on postoperative day 23. In the second case, sudden death occurred due to a pulmonary embolism, despite continued administration of proper antithrombotic prophylaxis. This patient had returned to the referring hospital, where he underwent an orthopedic procedure.

Complete coverage of the left subclavian artery (LSA) was necessary in nine patients (40.9%). Revascularization of the LSA was necessary in two cases (acute left arm ischemia in both patients). Prompt revascularization saved both limbs and no neurological complications occurred. In the remaining patients, there were no complications related to LSA coverage.

In all cases, TEVAR resulted in the complete exclusion of the injured section of the aorta, without endoleak. No re-intervention was necessary during the follow-up period. No neurological complications were observed.

### Aortic injury severity

There were two patients with type I injury (9.1%). Intimal damage of <1 cm was observed in an initial angio-CT scan of one patient, who was treated conservatively. In a subsequent angio-CT scan, the enlargement of the lesion site was observed, and the treatment strategy was changed. TEVAR was performed, resulting in the complete exclusion of the lesion. In the second case, the initial intimal injury was larger and TEVAR was performed in an emergency setting.

We did not observe any type II injuries (intramural hematoma).

Fifteen patients (68.1%) had type III injuries. Complete transection of the aorta (type IV injury) was recognized in 5 patients (22.8%) (Table 1). The most common co-existing injuries were limb fractures and pelvic fractures (*n* = 13; 59.1%). The concomitant injuries are listed in detail in Table 2. The injury severity score (ISS) was calculated in all cases. The scores ranged from 25 to 66 (43.2 ± 12.54).

**Table 1:** The characteristics of the patients with type III and IV injuries

	Type of injury		*p*
	III (*n* = 15)	IV (*n* = 5)	
Age	39.7 ± 12.1 (25–60)	27.2 ± 4.6 (22–33)	0.039
Aortic arch index (cm)	4.37 ± 0.64 (3.38–5.48)	3.12 ± 0.64 (2.3–3.59)	0.007
Aortic arch angle (degrees)	45.51 ± 5.61 (35.18–53.35)	36.29 ± 3.39 (33.07–44.8)	0.002
Injury severity score	43 ± 9.5 (25–57)	52 ± 14.1 (38–66)	0.035
Ascending aorta diameter (mm)	34 ± 3.68 (27–42)	30 ± 5.85 (22–38)	0.101
Aortic arch diameter (mm)	27 ± 2.69 (23–32)	21 ± 2.86 (18–25)	0.018
Descending aorta diameter—LSA (mm)	25 ± 3.58 (19–31)	20 ± 3.31 (16–24)	0.043
Descending aorta diameter—pulmonary bifurcation (mm)	26 ± 3.32 (20–31)	20 ± 3.27 (15–24)	0.035
Descending aorta diameter diaphragm (mm)	23 ± 3.07 (18–28)	18 ± 3.11 (14–22)	0.26
Mortality	2 (13.3%)	0	0.58
Male sex	11 (73.3%)	4 (80%)	0.56
ICU stay (mean)	10 ± 5.7 (1–23)	8 ± 5.5 (2–14)	0.50
Hospital stay(mean)	22 ± 35.2 (2–141)	25 ± 18.4 (18–60)	0.99
LSA coverage	10 (66.6%)	1 (20%)	0.24
Length of stent-graft (mm)	156 ± 28.4 (111–202)	130 ± 14.5 (113–147)	0.12

*ICU* intensive care unit, *LSA* left subclavian artery

**Table 2:** Concomitant injuries and adjunct procedures

Concomitant injuries and adjunct procedures	*n* = 22	%
Limb fractures	13	59.10
Pelvic fracture	13	59.10
Craniocerebral trauma	6	27.20
Rib fractures	9	40.90
Sternum fracture	2	9.10
Pleural cavity drainage	6	27.20
Laparotomy	5	22.70
Alcoholic intoxication (>1‰)	5	22.70

Because of the small sample size, it was only possible to analyze the patients with type III and type IV injuries. The ages of the patients with type III and type IV injuries ranged from 25 to 60 years (39.7 ± 12.08) and 22 to 33 years (27.2 ± 4.6), respectively (*p* = 0.039). The two groups did not differ in terms of gender; the type III injury group included 11 males and the type IV injury group included 4 males (*p* = 0.56).

### Anatomical considerations

The univariate analysis revealed a significant difference in the aortic diameter of the patients with type III and type IV injuries. The aortas at the level of the aortic arch in the patients with type III damage were significantly larger in comparison to the patients with type IV injuries. There were also significant differences in the diameter of the descending aorta at the level of LSA and pulmonary division, but not so at the level of a diaphragm (Table 1).

The aortic arch index and aortic arch angle were 6.59–7.08 cm (average 6.83 cm) and 55.2°–61.4° (average 58.3°), respectively, in the patients with type I injuries.

The aortic arch index values of the patients in the type III injury group ranged from 3.38 to 5.48 cm (4.37 ± 0.64 cm); in the type IV injury group they ranged from 2.3 to 3.59 cm (3.12 ± 0.64 cm). The difference was statistically significant (*p* = 0.007).

The aortic arch angle in the type III injury group ranged from 35.18° to 53.35° (45.51° ± 5.7°), while that in the type IV group ranged from 33.07° to 44.8° (36.29° ± 3.39°). This difference was statistically significant (*p* = 0.002) (Table 3).

**Table 3:** Assessment of the aortic arch according to the aortic index and aortic arch angle

Type of injury	Aortic arch index (cm)	Aortic arch angle (degrees)
I (*n* = 2)	6.83 ± 0.23 (6.59–7.08)	58.3 ± 2.7 (55.2–61.40)
III (*n* = 15)	4.37 ± 0.64 (3.38–5.48)	45.51 ± 5.7 (35.18–53.35)
IV (*n* = 5)	3.12 ± 0.64 (2.3–3.59)	36.29 ± 3.39 (33.07–44.8)

**Fig. 2:**
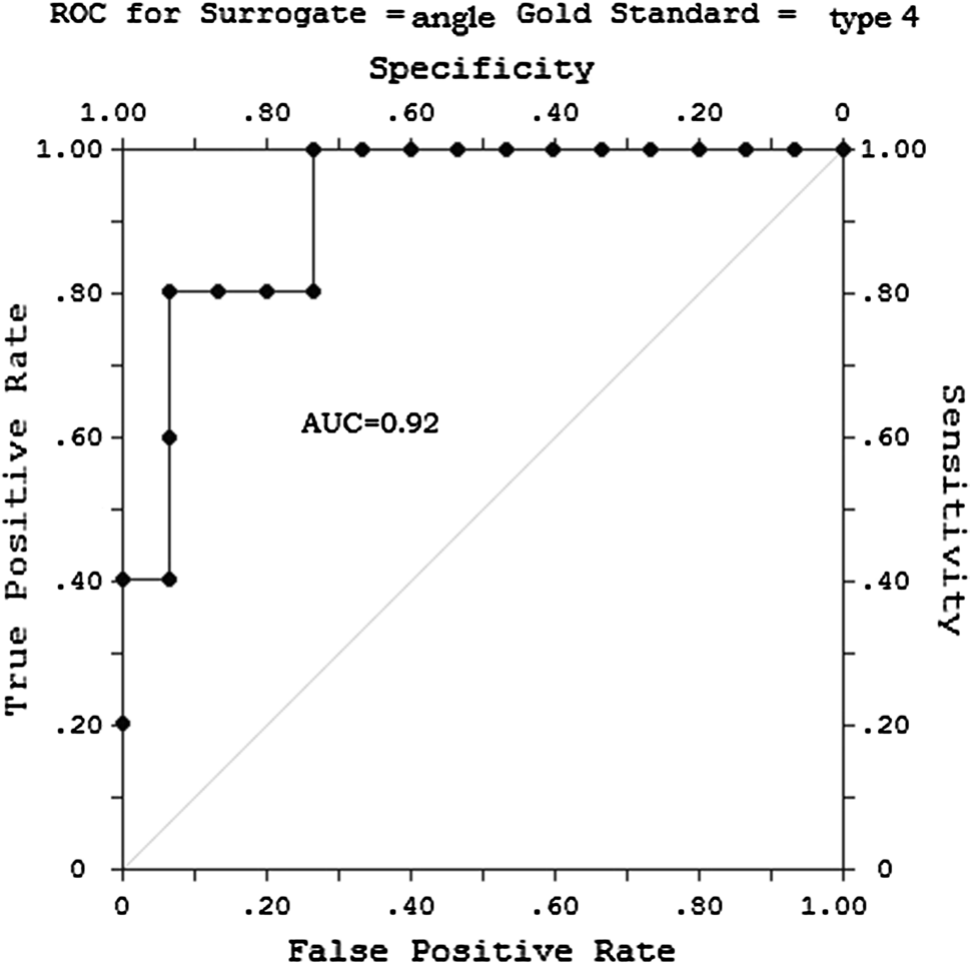
The ROC curve analysis for type IV injury. The cutoff aortic angle for predicting the occurrence of a type IV injury was ≤36.5°. Area under the curve = 0.92 (*p* = 0.045)

All of the variables that were identified as significant on the univariate analysis were included in a logistic regression analysis. Due to the low number of cases in each of the injury groups, each regression model could only include three variables. We, therefore, built several models. All of the models accounted for sex, age and one of the variables listed in Table 4. The aortic arch angle was the only variable that was found to be an independent risk factor for the occurrence of a specific type of injury in the logistic regression analysis (logistic regression, OR 1.5; 95% CI 1.03–2.2; *p* = 0.03 for the occurrence of a type III injury). The ROC curve analysis revealed that the critical aortic angle for the occurrence of a type IV injury was ≤36.5°. The area under the ROC curve was 0.92 (Fig. 2).

**Table 4:** The results of the logistic regression analysis

	Odds ratio	95% CI (confidence interval)	*p* Value
Aortic arch angle	1.5	1.03–2.2	0.03
Aortic diameter–ascending aorta level	1.148	0.821–1.604	0.4198
Aortic diameter–arch level	2.09	0.875–4.993	0.0969
Aortic diameter–diaphragm level	1.541	0.724–3.281	0.2623
Aortic diameter–PA level	1.994	0.903–4.406	0.0878
Aortic diameter–LSA level	1.339	0.862–2.08	0.1932
ISS	0.855	0.725–1.008	0.0615

*LSA* left subclavian artery, *PA* pulmonary artery, *ISS* injury severity score

## Discussion

Traumatic aortic injury is a life-threatening condition that requires prompt treatment. Teixer et al. investigated the autopsy examination results of blunt chest injury victims and noted that aortic injury was present in 1/3 of the cases, 67% of which had an injury of the aortic isthmus [11]. At the present time, endovascular treatment is a treatment of choice, with superior short-term results in comparison to open repair [12] because TEVAR minimizes surgical trauma to the already unstable patient [13].

The short-term mortality rate in our study was 9.1%. The technical success rate in the acute cases was 100%. Comparable data were presented by Rahim et al., Azizzadeh et al. and Yamaguchi et al. [14–16]. Coverage of the LSA was necessary in nine cases. In acute cases, this is a legitimate solution to obtaining a proximal landing zone of sufficient length to provide a perfect seal of the stent-graft and for preventing type Ia endoleak [17].

It is commonly believed that the direct force of the injury is responsible for the damage of the aorta. Other theories on this subject include the “water-hammer” effect, the “osseous pinch” effect, and aortic rupture due to a sudden increase in intraabdominal pressure [18, 19]. Each of these theories partially explains the causative mechanism of TAI; however, none explain the broad spectrum of injury presentations, which range from minimal intimal tears to the complete transection of the aorta.

The aim of this study was to evaluate whether there is a relationship between the aortic anatomy and the degree of aortic injury. To answer this question, we propose the use of the evolution of previously described parameters that may influence the severity of aortic trauma. These measurements are based on angio-CT reconstruction.

The first parameter, the aortic arch index, was obtained by measuring the distance from the outer wall of the ascending aorta to the outer wall of the descending aorta on the lesser curvature on the horizontal plane, at the height of mid-left bronchus. This represents the evolution of a parameter suggested by Alberta et al. [20] who compared the curvature of the aortic arch radius in aortic trauma patients with that in aortic aneurysm patients. They assessed their parameter in axial angio-CT images by measuring the distance from the inner wall of the ascending aorta to the inner wall of the descending aorta at the level of the pulmonary artery division. Half of this measurement is the estimated radius of the curvature of the aortic arch. The radius values of trauma patients were found to be significantly lower. In the present study, we found that the aortic arch index values of type IV injury patients were significantly lower than the values of type III injury patients. Patients with grave aortic injury (the complete transection of the aorta) had lower aortic arch index values.

The second parameter is an evolution of work described by Agnoletti et al. [21], who proposed the measurement of the arch angle in lateral aortic arch aortography. The angle between the center of the ascending and descending aorta was measured at the level of the division of pulmonary artery, and the highest point of the aortic arch. Measurements made in aortography—particularly assessments based on lateral projections—may be inaccurate when reconstructions are not created using the modern software programs that are used in the evaluation of angio-CT pictures. In the present study, we used an evolution of this parameter, but it was based on vascular computed tomography reconstruction. We named the parameter the aortic arch angle. We measured the angle of the arch between the two aforementioned points and the highest point of the aortic arch in oblique MPR reconstruction.

The study population only included 2 patients with type I aortic injuries; thus, it was not possible to include them in the statistical analysis. However, both of the patients with this minor type of injury—in which conservative treatment may be considered—had a mild aortic arch angle. We had no patients with type II injuries. This observation is in line with the observations of other authors. Many authors question the inclusion of intramural hematoma injury in aortic injury scales, and alternative aortic injury scales, in which this type of injury is omitted, have been suggested [22, 23]. The univariate analysis revealed significant differences in the age of the patients, the aortic diameter, and the severity of injury (according to the ISS) between the patients with type III and IV injuries. The univariate analysis also revealed a significant difference in the aortic arch index values and in aortic arch angles of the type III and type IV cases. However, the aortic arch angle was the only independent risk factor that influenced the severity of aortic injury in the logistic regression analysis.

Several authors who investigated the results of TEVAR for TAI have mentioned problems in the treatment of patients with a small aortic diameter or an acute aortic arch angle. The problem is especially common in young patients. This problem may be overcome by selecting stent-grafts that allow for the perfect conformability of the proximal section of the stent-graft. Few reports have described the remodeling of the aortic arch in association with age. It has been proven that the diameter of the aorta as well as the length of aortic arch and the ascending aorta increase with age [4–6]. Chiu et al. proposed an aortic arch measurement [24]. It requires the designation of a central line and the application of the points of origin of the aortic arch branch vessel. It reflects the changes that occur in the origins of the branch vessel from the arch due to aging. However, thus far there have been no reports of a parameter that reflects the changes in the geometry of the aortic arch that occur in association with aging. One may wonder whether the higher incidence of severe aortic injury in young patients may be related to the acute aortic arch angles that are often encountered in these patients.

The present study is associated with some limitations, including the retrospective nature of the study, the use of different CT scanners, and the potentially arbitrary placement of the measurement points on the CT scans (selection of mid-left bronchus level may produce inter-observer inconsistency). The low number of patients is also a serious limitation and the data should be further validated in larger cohorts.

There is an apparent need to extend the understanding of the biomechanics of the aortic arch in the current surgical era, in which endovascular devices are increasingly used to treat pathological conditions in this area of aorta. The shape of the aortic arch has an unquestionable influence on the occurrence of endoleak, bird-beaking, and even on the collapse of stent-grafts in TEVAR [7, 25, 26].

## Conclusions

The low number of complications in the endovascular treatment of injuries of the aortic isthmus indicates that it is safe and effective. The severity of TAI is influenced by the sharpness of the aortic arch. There is an inverse relationship between severity of aortic injury and the aortic arch index.
